# Housing status and accidental substance-related acute toxicity deaths in Canada, 2016–2017

**DOI:** 10.24095/hpcdp.44.7/8.03

**Published:** 2024-07

**Authors:** Amanda VanSteelandt, Raahyma Ahmad, Aganeta Enns, Beth Jackson, Tanya Kakkar, Fiona Kouyoumdjian

**Affiliations:** 1 Substance-Related Harms Division, Public Health Agency of Canada, Ottawa, Ontario, Canada; 2 The Canadian Association of People Who Use Drugs, Dartmouth, Nova Scotia, Canada; 3 Health Equity Policy Division, Public Health Agency of Canada, Ottawa, Ontario, Canada; 4 Ontario Ministry of Health, Toronto, Ontario, Canada

**Keywords:** drug overdose, opiate overdose, poisoning, mortality, homeless persons, unhoused, unsheltered, homelessness

## Abstract

**Introduction:**

There is a complex relationship between housing status and substance use, where substance use reduces housing opportunities and being unhoused increases reasons to use substances, and the associated risks and stigma.

**Methods:**

In this descriptive analysis of people without housing who died of accidental substance-related acute toxicity in Canada, we used death investigation data from a national chart review study of substance-related acute toxicity deaths in 2016 and 2017 to compare sociodemographic factors, health histories, circumstances of death and substances contributing to death of people who were unhoused and people not identified as unhoused, using Pearson chi-square test. The demographic distribution of people who died of acute toxicity was compared with the 2016 Nationally Coordinated Point-In-Time Count of Homelessness in Canadian Communities and the 2016 Census.

**Results:**

People without housing were substantially overrepresented among those who died of acute toxicity in 2016 and 2017 (8.9% versus <1% of the overall population). The acute toxicity event leading to death of people without housing occurred more often in an outdoor setting (24%); an opioid and/or stimulant was identified as contributing to their death more frequently (68%–82%; both contributed in 59% of their deaths); and they were more frequently discharged from an institution in the month before their death (7%).

**Conclusion:**

We identified several potential opportunities to reduce acute toxicity deaths among people who are unhoused, including during contacts with health care and other institutions, through harm reduction supports for opioid and stimulant use, and by creating safer environments for people without housing.

**Highlights**

- 8.9% of people who died of accidental substance-related acute toxicity in 2016 and 2017 were unhoused at the time of their death compared to less than 1% of the general population in Canada.
- One in four of the acute toxicity events that lead to death occurred outdoors.
- People who were unhoused at the time of their death had an opioid and/or stimulant identified as contributing to their death more often than those with housing.
- Toxicology tests detected opioids and stimulants in combination in more than half of the people who were unhoused at the time of their death.

## Introduction

The overdose crisis in Canada is a significant public health concern, with 36 442 deaths related to apparent opioid toxicity between January 2016 and December 2022.^1^ Provincial and municipal reports show a greater impact on some populations than others, including people who are unhoused.^2^-^5^ An estimated 235000 people were unhoused in Canada in 2016, including 22190 people who were in shelters on any given night.^6^,^7^

In this paper, we have chosen to use “people who were unhoused” or “people without housing” rather than “people who were experiencing homelessness.” A house is a physical shelter, but a home encompasses more than a physical location and is tied to personal meanings and social connections.^8^-^11^ A person without access to a stable or safe physical shelter may still have a home in the people around them, the spaces they live in and wider community.

People who are unhoused have higher rates of substance use than the general population.^12^,^13^ The relationships between housing insecurity (“the loss of, threat to, or uncertainty of a safe, stable, and affordable home environment”^14^^,p.344^), substance use and harms from substance use are complex. Substance use and substance use disorders are commonly cited reasons for housing loss, and people who are unhoused are likely to have experienced trauma, mental illness and/or incarceration, which contribute to a higher risk of substance use.^15^-^19^ Being unhoused may increase use as a way of dealing with the difficulties and dangers of life without secure, private housing.^15^,^20^-^22^ In addition, being unhoused directly and indirectly increases the harms associated with substance use, including acute toxicity events.^23^ Due to stigmatization, as well as logistical barriers to service access, people who are unhoused may have worse access to treatment and harm reduction services.^24^ They may also feel the need to conceal or rush substance use, use alone and use larger amounts to avoid drug possession charges.^25^,^26^ Finally, being unhoused may put people in situations that are criminalized, and periods of incarceration may disrupt continuity of treatment and services as well as drug supply, contributing to increased risk of acute toxicity events upon release, in part because of reduced drug tolerance.^27^-^29^

The aim of this study is to describe the sociodemographic profiles, health history, substances involved and the circumstances of death due to accidental substance-related acute toxicity of people who were unhoused.

## Methods

***Ethics statement***

This study was reviewed and approved by the Public Health Agency of Canada Research Ethics Board (REB 2018-027P), the University of Manitoba Health Research Ethics Board (HS22710) and the Newfoundland and Labrador Health Research Ethics Board (20200153).

***Main data source***

This study uses data from a national, retrospective chart review of substance-related acute toxicity deaths from coroner and medical examiner investigation files from between 1 January 2016 and 31 December 2017. Although death investigation procedures vary across Canada, the death investigation files generally contain some combination of a death certificate, coroner or medical examiner report, witness statements, medical records, police reports, toxicology reports and/or autopsy reports. 

The case definition includes all individuals who died in Canada between 1 January 2016 and 31 December 2017 of accidental acute toxicity resulting from the direct effects of the administration of exogenous substance(s), where one or more of the substances was a drug or alcohol. Variables collected from the death investigation files by the data abstractors included sociodemographic risk factors, documented substance use and medical history, circumstances of death and toxicological findings. Because histories of mental and physical health conditions or symptoms were collected from medical records or witness statements in the death investigation files, these conditions and symptoms are not necessarily clinical diagnoses. Similarly, the study only captured what was available in the file, which might not have included the person’s entire medical history or life experiences.

Abstractors received training and written guidance on what kinds of information to look for in death investigation files and how to code or describe this in the database. Potentially traumatic events might include: a friend’s or family member’s health problem; intimate partner problem (e.g. divorce, discord) or other relationship problem (e.g. family argument); job- or school-related problem; financial problem; recent death by suicide of a friend or family member; other death of a friend or family member; criminal legal problem (e.g. arrest, jail, court case) or other legal problem (e.g. custody dispute, civil law); interpersonal violence (as victim or perpetrator); child maltreatment experience; foster care experience; residential school experience; or experience of sexual abuse or physical abuse or assault. An abstractor might also have entered another event with an explanation for how it meets the definition of a potentially traumatic event. The chart review study protocol, database and definitions of variables are described in greater detail elsewhere. 30

***Definitions for housing status***

To identify people who were unhoused, this study uses the Canadian Observatory on Homelessness’ definition of homelessness, as “the situation of an individual … without stable, permanent, appropriate housing, or the immediate prospect, means and ability of acquiring it.”^31^^,p.1^ This includes people living unsheltered on the street, staying in emergency shelters and temporarily accommodated by couch surfing, staying with friends or family or trading informal employment or resources for housing. It also includes people at immediate risk of being unhoused because of job loss or eviction by a property owner, for example.

People who died of accidental acute toxicity in the national dataset were identified as “unhoused at the time of death” and/or “unhoused within 6 months of death” based on variables related to their living arrangements, any recent moves within 6months of death, and open text comments abstractors made about the investigation file. A specific variable in the database indicates evidence in the death investigation file that the person experienced housing instability in their lifetime. We categorized those who had no documented evidence of being unhoused at any point in their lifetime (i.e. are not included in any of the variables for being unhoused) as “not identified as being unhoused.” As coroner and medical examiner files are not a complete record of a person’s life, some people categorized as “not identified as being unhoused” might be misclassified. 

People who were hospitalized or in a correctional facility or other institution at the time of death were excluded from both “unhoused at time of death” and “not identified as being unhoused” categories, and are not included in comparisons between these two groups. Data on living arrangements were missing for 10.9% of all people; they too were excluded from further analysis. Comparisons of people missing data on their living arrangement with those not missing data revealed statistically significant differences in a subset of key variables that included age, sex, substance use history, history of substance use disorder (excluding alcohol) and contact with the health system in the year before death.

***Statistical analyses***

We selected variables for analysis based on hypothesized relationships with housing and substance use or as potential intervention points. As noted previously, death investigation protocols vary across jurisdictions; as a result, many of the variables were not available in the source material for all jurisdictions, limiting descriptive analysis to the minimum numbers and proportions of people who died of acute toxicity who had information recorded for a given variable. We conducted Pearson chi-square tests to assess statistical differences between people who were unhoused at the time of their deaths and people not identified as unhoused (*p*< 0.05). As this study is descriptive and the variables were preselected based on hypothesized relationships, no adjustments were made for multiple comparisons. The substances and substance combinations most frequently contributing to the deaths of people in both subpopulations were identified using the ComplexUpset package.^32^

We also compared the demographic distribution of both populations who died of acute toxicity with the 2016 Coordinated Point-In-Time Count of Homelessness in Canadian Communities^20^ and the 2016 Census.^33^ Between 1 January 2016 and 30April 2016, 32 communities across Canada participated in a coordinated count of people in shelters and on the streets within their community. Some of the counts also included people who were in health care or correctional facilities and had no place to go on discharge. The census provides a statistical overview of the population of Canada every 5 years. Individuals are counted at their usual place of residence, which can be a private or collective dwelling. While collective dwellings include shelters, the census is limited in its ability to capture people without housing.^12^ Tests of statistical difference were not used in these comparisons because the populations from these three data sources are not independent.

To protect privacy, all counts from the chart review study data are randomly rounded to base 3, and proportions and rates are based on rounded counts.^30^ All statistical analyses and random rounding were performed using R statistical software^34^ and RStudio (version 2022.02.0).

## Results

***Comparison with the general population***

**Figure 1:**
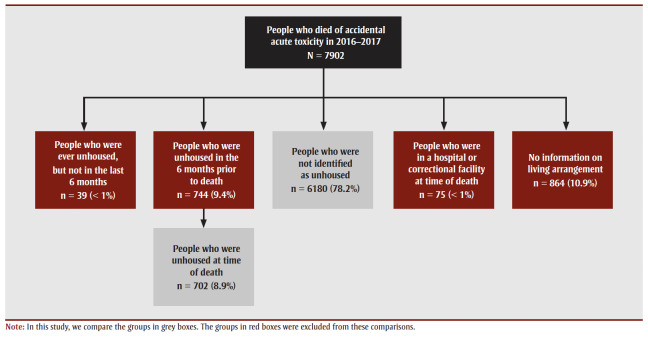
Housing status of people who died of accidental acute toxicity, Canada, 2016–2017

Based on the available data for 7902 people in Canada who died of accidental acute toxicity in 2016 or 2017, at least 9.4% (n = 744) had been unhoused within 6 months of their death, and 8.9% (n=702) were unhoused at the time of their death (Figure 1).

As 0.06% to 0.10% of the population of Canada was unhoused on a given day in 2016, with 0.67% unhoused at any time in 2016,^11^,^12^,^33^ people who were unhoused were overrepresented among those who died of accidental acute toxicity.

Of the people who died of accidental acute toxicity, those who were unhoused tended to be younger (20–49 years; *p*<0.001) and male (*p* < 0.05) (Table 1). Compared with the overall population^33^or unhoused population in Canada in 2016,^21^ people who died of accidental acute toxicity and were unhoused were more commonly aged between 30 and 59 years and more often male (Table 1).

**Table 1:** Distribution by sex and age groups of the people who died of accidental acute toxicity, by housing status at time of death, 2016–2017, and for the total population, Canada, 2016

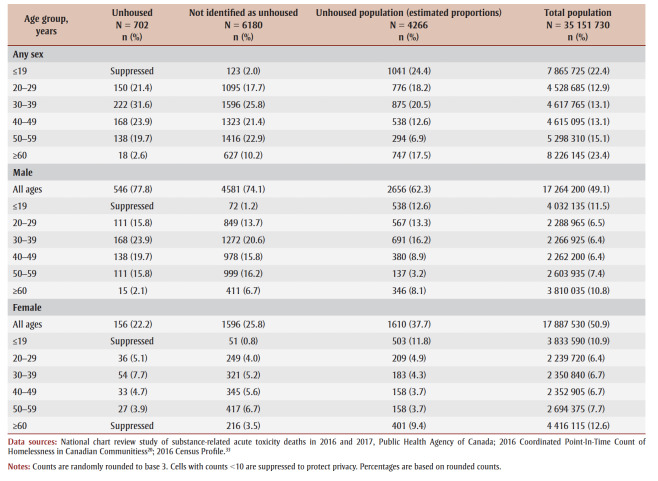

***Encounters with the health care system by housing status***

The minimum proportion of people who had contact with the health care system in the year before their death from accidental acute toxicity was higher for people not identified as unhoused than for those without housing (75% vs. 68%; *p*<0.001). People without housing accessed outpatient and inpatient services more often than those not identified as unhoused (Table 2). They also sought care due to acute injury (8% vs. 4%; *p* < 0.001), a nonfatal acute toxicity event (14% vs. 7%; *p* < 0.001) or substance use and/or addictions (16% vs. 12%; *p*<0.05) more often. However, the reason for seeking care was unknown for many people, irrespective of their housing status, and differences between the two groups may be related to reporting biases rather than true differences.

**Table 2:** Distribution of health care system encounters for people who died of accidental acute toxicity, by housing status at time of death, Canada, 2016–2017 (N = 7902)

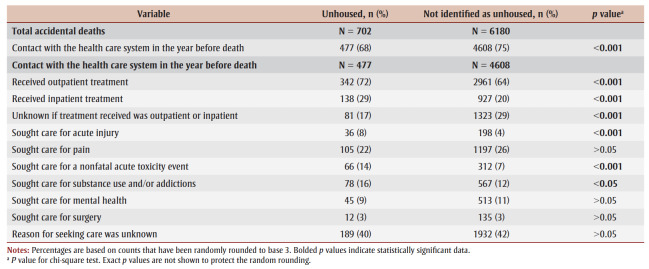

***Histories of substance use, mental health and potentially traumatic life events***

Of the people who died of accidental acute toxicity, those without housing had histories of substance use and of chronic substance use more often than those not identified as unhoused (92% vs. 83% and 63% vs. 54%, respectively; *p* < 0.001) (Table 3). The proportions of people with histories of substance use disorder or alcohol use disorder were similar for both populations. Depression or depressive symptoms were recorded more often for people not identified as unhoused than for those without housing (26% vs. 15%; *p*< 0.001). Mental health history was unknown for 29% of people without housing and 22% of those not identified as unhoused (*p*<0.001).

**Table 3:** Distribution of health history, circumstances of death and substances contributing to death of people who died of accidental acute toxicity, by housing status at time of death, Canada, 2016–2017 (N = 7902)

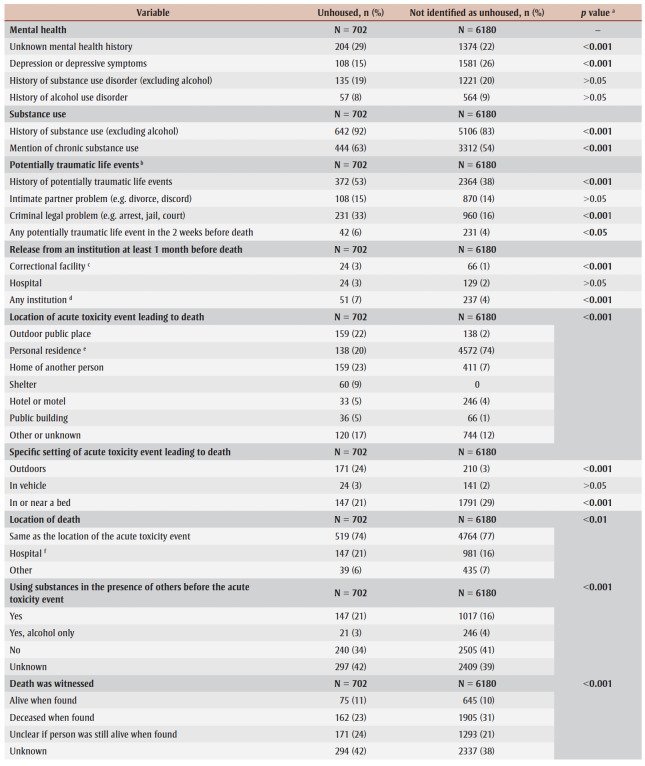 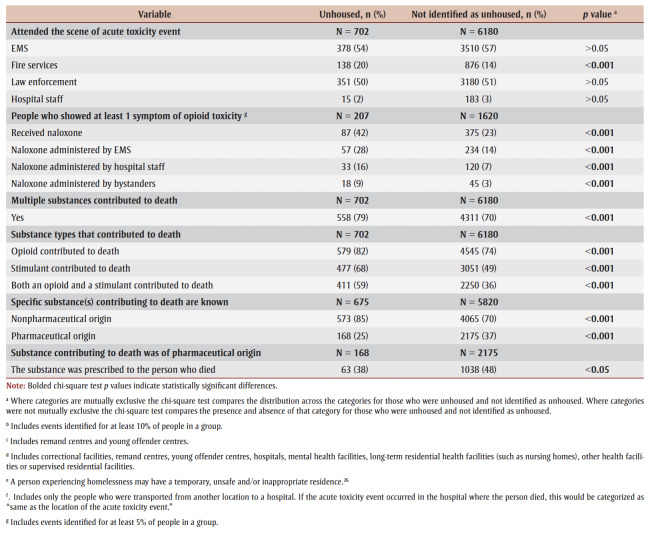

Among the people who died of accidental acute toxicity, about half (53%) of those without housing had a history of at least one potentially traumatic event compared with about one-third (38%) for people not identified as unhoused, a difference of 17percentage points (*p*<0.001) (Table 3). People without housing experienced a potentially traumatic life event in the 2weeks before their death more commonly than peers who were not identified as unhoused (6% vs. 4%; *p* < 0.05). 

While criminal legal problems and intimate partner problems were the most frequently identified potentially traumatic life events for both populations, criminal legal problems were significantly more common for people who were unhoused (33% vs. 16%; *p* < 0.001). 

***Recent institutionalization***

At least 7% of people who died of accidental acute toxicity and were unhoused had been discharged from an institution up to one month before their death (*p*<0.001). The proportion discharged from a correctional facility was higher for people without housing than those not identified as unhoused (3% vs. 1%; *p*<0.001), but the proportions discharged from a hospital were similar (Table 3).

***Circumstances of death***

The locations of the acute toxicity events that led to accidental deaths differed significantly by housing status (Table 3). For people without housing, the acute toxicity events most often occurred in an outdoor public place, personal residence or home of another person (about 20%–23% of acute toxicity deaths for each location). For people not identified as unhoused, most fatal acute toxicity events occurred in a personal residence (74%). About one in four acute toxicity events experienced by people without housing occurred outdoors (for example, in outdoor public places, front or back yards of residences, sidewalks beside buildings), compared to only 3% for people not identified as unhoused (*p* < 0.001). People not identified as unhoused more commonly experienced the acute toxicity event in or near a bed (29%; *p*<0.001). 

Regardless of housing status, the location of death was the same as the location of the acute toxicity event for most of the people who died, but people not identified as unhoused more often died at the same location and people without housing more often died in hospital after transport from another location.

Where data were available, the majority of people, irrespective of housing status, were using substances alone prior to the acute toxicity event, but people without housing were significantly more likely to be in the presence of other people (21%; *p*< 0.001) (Table 3). 

People without housing who were found exhibiting one or more symptoms of opioid toxicity (i.e. snoring/gurgling, difficulty breathing, pinpoint pupils, unconscious/unresponsive or blue lips/fingernails/face) were more likely to have received naloxone than people not identified as unhoused (42% vs. 23%; *p* < 0.001). Among those who exhibited at least one symptom of opioid toxicity, people without housing were more likely to have received naloxone from EMS (28% vs. 14%), hospital staff (16% vs. 7%) and bystanders (9% vs. 3%), compared to people not identified as unhoused (*p*<0.001). EMS, law enforcement and hospital staff were equally likely to attend the scene for people with either housing status, but fire service personnel were more likely to attend the scene for people without housing (20%; *p* < 0.001). 

While the proportions of individuals known to still be alive when found were similar in both groups, a greater proportion of people not identified as unhoused were already dead when found (31% vs. 23%). For a greater proportion of people without housing, it was unclear or unknown whether they were still alive when found.

***Substances contributing to death***

Multiple substances contributed to most deaths, but deaths involving multiple substances were significantly more common for people without housing than for those not identified as unhoused (79% vs. 70%; *p*<0.001). Where the specific substance or substances contributing to death were known, their origin was less often pharmaceutical in nature (25% vs. 37%; *p*<0.001) and less often prescribed to the person who died (38% vs. 48%; *p*<0.05) for people without housing (Table 3).

The substances most commonly contributing to accidental toxicity deaths were the same irrespective of housing status, but they differed by degree of contribution (Table 4). Fentanyl most frequently contributed to death for people with either housing status, but contributed significantly more frequently for people without housing than for those not identified as unhoused (*p* < 0.001). Cocaine contributed to a similar proportion of deaths for people with both housing statuses, but other stimulants like methamphetamine and amphetamine contributed to a greater proportion of deaths for people without housing than those with housing (*p*<0.001).

**Table 4:** Origin and contribution of other substances for substances that contributed to at least 10% of accidental deaths, by housing status at time of death, Canada, 2016–2017 (N = 7902)

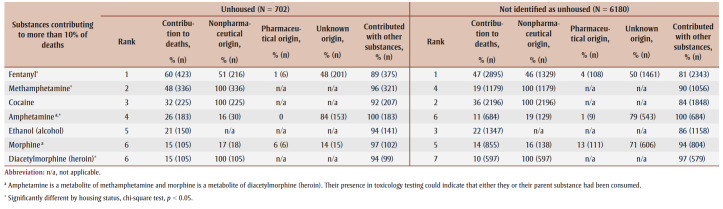

While fentanyl was most often involved in the substance combinations that most frequently contributed to death for people with both housing statuses, the involvement of stimulants varied ( Table 5). For people without housing, methamphetamine was more common among the highest-ranking substance combinations contributing to death, while cocaine was more common for people not identified as unhoused.

**Table 5:** Exclusive substances and substance combinationsa contributing to most of the accidental acute toxicity deaths, by housing status at time of death, Canada, 2016–2017 (N = 7902)

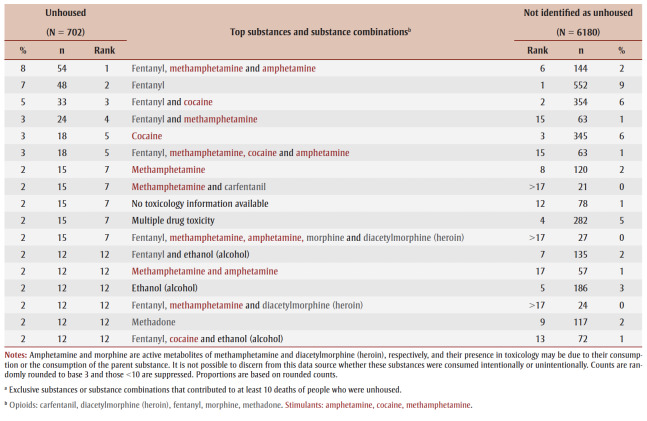

## Discussion

People without housing are substantially overrepresented among those who died of acute toxicity in 2016 and 2017 (8.9% of people who died were unhoused compared to <1% of the overall population of Canada). The study findings suggest several opportunities for intervention and improving supports for people who are experiencing or at risk of being unhoused and are at risk of an acute toxicity death.

At least 7% of people without housing, which is about twice as many people not identified as unhoused, had been discharged from a health care or correctional institution in the month preceding their death. The stays in the institutions may or may not have been related to substance use. Risk of acute toxicity death is higher after a recent discharge for many reasons: a person’s tolerance decreases after a period of not using substances; the stay could have interrupted access to and continuity of treatments and supports (e.g. opioid agonist therapies) and, depending on the length of stay, they may be experiencing withdrawal, leading to higher-risk use; or the available substances and their toxicity could have changed.^35^-^37^

Hospitals and correctional facilities could strengthen their transition planning prior to discharge and connect people experiencing or at risk of being unhoused with evidence-based harm reduction, treatment, health care and housing services to prevent acute toxicity events. A high proportion of people without housing had contact with the health care system more broadly prior to death (through outpatient or inpatient care); these encounters provide opportunities to connect individuals to necessary health and social services.

While people who were unhoused and died of acute toxicity had known histories of substance use more often than people not identified as unhoused, their histories of substance use disorders and of any mental health conditions were similar. This finding may be because of a lack of recorded history for people who were unhoused due to limited access to health services or limited information available during death investigations. One in every two people without housing who died had a documented history of at least one potentially traumatic event during their lifetime, which was significantly higher than for people with housing. This highlights the need for accessible, inclusive and trauma-informed services, consistent with Canadian clinical guidelines, for the complex health and social circumstances of people without housing.^38^

For people not identified as unhoused, the acute toxicity event occurred in a personal residence three times out of four. Among people without housing, one-quarter of the acute toxicity events occurred in an outdoor setting. Substance use in an outdoor setting might increase the odds of a witness noticing a medical emergency; however, it may also result in practices to conceal use (rushing use or using larger amounts) to avoid law enforcement encounters and drug possession charges, and use in outdoor or public places has been associated with increased risk of experiencing an acute toxicity event.^25^,^26^,^39^

While people with both housing statuses were more often using substances alone prior to the acute toxicity event, people without housing were more likely than those not identified as unhoused to be using substances in the presence of others, presenting a higher potential for intervention. Harm reduction programs, housing services and law enforcement policies could promote safer environments for both shelter and substance use, where medical assistance and naloxone are more readily accessible. Improving service integration and availability of wraparound services within existing supports (e.g. shelters/housing services, harm reduction and treatment programs) may also reduce harms and improve outcomes for people without housing, who can have complex and interrelated health and social service needs. 

The pattern of substances involved in death differed by housing status. Stimulants and opioids are accessible street drugs and use of these substances to cope with trauma and situational stressors has been described previously.^40^,^41^ Use of stimulants such as methamphetamines may help people stay awake and alert when they are unsheltered and unsafe.^42^,^43^ The several-hour longer half life and generally lower price may explain why methamphetamines, rather than cocaine, more commonly contributed to the death of people experiencing homelessness; cocaine was more commonly a contributor to death of people with housing.^44^ Co-use of opioids and stimulants has been reported to calm down a person after using a stimulant, to alleviate withdrawal symptoms or paranoia from a stimulant, to avoid feeling drowsy when using an opioid, to create a pattern of successive stimulation and sedation or to balance the effects of each substance.^45^ Alternatively, the presence of both these substances may be unintentional and a result of contamination. Consulting with people who are or at risk of being unhoused about the substances they use and their patterns of use could inform health promotion and harm reduction services, including safer supply options. Knowing what substances are causing harm can also help tailor training for first responders and bystanders responding to acute toxicity events.

***Strengths and limitations***

Both the national and chart review study estimates of unhoused people are based on point-in-time counts (for national estimates, it is the day of the count; for the chart review study, it is the day of death), and do not include all people who are unhoused in a community over a period of time. People often cycle in and out of being unhoused, and those who are temporarily staying with friends or family were less likely to be identified during the count.^46^,^47^ People who are experiencing housing insecurity or who are at immediate risk of being unhoused were also less likely to be identified during the count.

The purpose of death investigations is to establish the cause and manner of death and, in some cases, to provide recommendations to prevent deaths of a similar nature in the future. Therefore, coroners and medical examiners are not seeking some of the variables of interest to this study. Death investigation protocols and methods of data collection and the availability of certain variables vary across the country. For example, binary sex was always available, but gender identity, rarely. In addition, less information may be collected during death investigations of people who were unhoused because it can be difficult to identify witnesses, friends, family members or service providers who can speak to the personal histories of those who died. In death investigation files, it is not always clear whether someone was living with a friend or family member because of housing insecurity. Being unhoused, histories of substance use, mental health conditions and symptoms, and potentially traumatic experiences are all likely underreported in the chart review study; thus, the data represent the minimum proportions of people who had these experiences. The differences between people who were unhoused at the time of their death and those not identified as being unhoused may be underestimated due to misclassification of people if information was absent from the death investigation.

## Conclusion

This study identifies potential opportunities to reduce accidental acute toxicity deaths among people who are unhoused, including during contacts with health care services and through strengthening transition planning prior to release from institutions, taking into account the need for accessible, inclusive and trauma-informed services and improving service integration within existing supports, creating safer environments for shelter and substance use, and tailoring health promotion and harm reduction services to their specific needs. 

Since the study period, the COVID-19 pandemic has contributed to an increase in the number of people who are unhoused and increased many barriers to services for them.^48^ Research on the current relationships between housing status and substance-related harms and engagement with people with lived and living experience of being unhoused would be valuable to advance policies and programs to prevent further accidental acute toxicity deaths.

## Acknowledgements

We would like to acknowledge our collaborators at the offices of chief coroners and chief medical examiners across Canada for providing access to their death investigation files. We would also like to thank our co-investigators for their contributions in the development of the national chart review study on substance-related acute toxicity deaths: Matthew Bowes, Songul Bozat-Emre, Jessica Halverson, Dirk Huyer, Graham Jones, Jennifer Leason, Regan Murray, Erin Rees, Jenny Rotondo and Emily Schleihauf. 

## Funding

This study was funded by the Public Health Agency of Canada.

## Conflicts of interest

None to declare.

## Authors’ contributions and statement

AV: Methodology, conceptualization, writing – original draft, writing – review & editing, supervision, project administration, data curation, formal analysis, investigation

BA: Methodology, conceptualization, writing – review & editing

RA: Methodology, conceptualization, writing – original draft, writing – review & editing, data curation, formal analysis, investigation

AE: Methodology, conceptualization, writing – original draft, writing – review & editing, data curation, formal analysis

BJ: Methodology, conceptualization, writing – review & editing

TK: Methodology, conceptualization, writing – original draft, writing – review & editing, data curation, formal analysis, investigation

FK: Methodology, conceptualization, writing – review & editing

The content and views expressed in this article are those of the authors and do not necessarily reflect those of the Government of Canada or of the data providers.
